# Challenges in recruiting subjects to a pilot trial of patient-managed in-hospital insulin

**DOI:** 10.1186/s13104-015-1480-6

**Published:** 2015-10-01

**Authors:** Emily K. Acton, Charles E. Leonard, Mark H. Schutta, Serena Cardillo, Andrea B. Troxel, Rebecca Trotta, Sean Hennessy

**Affiliations:** Department of Neurology, Hospital of the University of Pennsylvania, Philadelphia, PA USA; Center for Clinical Epidemiology and Biostatistics, Department of Biostatistics and Epidemiology, Perelman School of Medicine at the University of Pennsylvania, Philadelphia, PA USA; Center for Pharmacoepidemiology Research and Training, Perelman School of Medicine at the University of Pennsylvania, Philadelphia, PA USA; Penn Rodebaugh Diabetes Center, Division of Endocrinology, Diabetes, and Metabolism, Department of Medicine, Perelman School of Medicine at the University of Pennsylvania, Philadelphia, PA USA; Department of Nursing, Hospital of the University of Pennsylvania, Philadelphia, PA USA; Department of Systems Pharmacology and Translational Therapeutics, Perelman School of Medicine at the University of Pennsylvania, Philadelphia, PA USA

**Keywords:** Patient self-managed insulin, In-hospital, Diabetes management, Pilot trial

## Abstract

**Background:**

To examine the feasibility of implementing clinician-supported inpatient self-managed insulin to aid in the planning of a randomized clinical trial.

**Results:**

We conducted a proof-of-concept interventional study of inpatients with diabetes mellitus who had hospital orders for basal-bolus or sliding scale insulin. Patients meeting inclusion criteria were offered the opportunity to manage their own basal-bolus insulin with support from a diabetes nurse practitioner. Over a three-month screening period, we conducted 361 screens in 336 patients, only eleven of whom met all inclusion criteria. None of these eleven eligible patients elected to enroll. The most common reason for refusal was lack of interest in self-managing insulin while acutely ill (36 %).

**Discussion:**

Future studies of patient-managed in-hospital insulin should consider enrolling less acutely ill patients with longer anticipated lengths of stay.

Trials registration: NCT02144441

## Background

Many patients with diabetes are adept at managing their home insulin regimen. When such patients become hospitalized in the U.S., however, their insulin is managed by clinical staff who may not always use best practices to prescribe, administer, and monitor insulin. The poor quality of in-hospital insulin management [1] is documented by figures showing that hypoglycemia and hyperglycemia occur during 5.7 and 32 % of non-intensive care unit patient-days, respectively [2]. Patient-directed care is gaining acceptance in many areas of medicine, including diabetes care. [3] The American Diabetes Association states that “[d]iabetes self-management in the hospital may be appropriate for competent youth and adult patients” [4]. Moreover, with the high rates of adverse effects and the potential for prolonged length of stay due to ineffective insulin management by staff, if patient-managed insulin could prove to be more effective, it might reduce the cost of hospital care for diabetes patients [5]. While patient management of in-hospital insulin is widespread in the UK, [5] few if any U.S. hospitals allow patients to manage their own non-pump insulin, and no randomized clinical trials (RCTs) have evaluated this approach.

We therefore began planning a RCT to examine the safety and effectiveness of patient-managed in-hospital insulin versus hospital-managed insulin. To assess the feasibility of such a trial, we performed a proof-of-concept pilot trial to answer the following questions: (1) What proportion of hospital inpatients receiving subcutaneous insulin meet inclusion criteria? (2) What proportion of eligible patients are willing to participate? and (3) What proportion of subjects are able to continue insulin self-management throughout their hospital stay?

## Results

### Methods

We designed a pilot trial with an intervention group but no control group. The study was conducted from July to November 2014 at the Hospital of the University of Pennsylvania. Enrollment was originally planned to be open for 6 months, but due low enrollment, was terminated after 3 months. We originally started recruiting on a single medical unit, but because of a low enrollment expanded to two additional medical units and one surgical unit.

The intervention was to allow eligible inpatients to manage their own basal-bolus insulin regimen with support from diabetes nurse practitioners (DNPs). Written informed consent would have been obtained from all subjects. Physicians would order insulin glargine (Lantus) vials and insulin aspart (NovoLog) flexpens without specifying doses. Subjects would have medication lockboxes placed at their bedside containing glargine vials, aspart pens, dextrose gel, a glucometer, and other necessary blood glucose management supplies. A DNP would meet daily with each subject to develop and monitor their basal-bolus regimens. Subjects would be permitted to perform their own finger-stick blood glucose measurements, administer their own insulin, and document glucometer readings and insulin doses on bedside flow sheets that would be reviewed by clinical staff and incorporated into the electronic medical record.

Inclusion criteria were: (1) admission or transfer to a participating floor in the 3 days prior to screening (to accommodate the lack of screening on weekends); (2) age ≥18 years; (3) diagnosis of type-1 or 2 diabetes mellitus; (4) a home basal-bolus insulin regimen administered via subcutaneous injection rather than pump; (5) willingness to use specified basal-bolus insulins (aspart pen and glargine vial); (6) most recent glycosylated hemoglobin of <7.5 % (<58 mmol/mol) measured in the 180 days prior to the current hospitalization; (7) active order for basal-bolus or sliding scale insulin; (8) willingness to document self-measured blood glucose results, self-administered insulin doses, food intake, and physical activity; and (9) approval from the clinical team. Exclusion criteria were: (1) order for an insulin infusion or pump; (2) inability to perform physical activities necessary for the study; (3) primary reason for current admission was related to glucose control or a medical condition that would impair the patient’s ability to manage insulin; (4) current order for a newly-prescribed corticosteroid or increased dose of a previously-prescribed corticosteroid; (5) enteral or parenteral nutrition; (6) expected length of stay <2 days; (7) risk for self-harm; (8) pregnancy; (9) inability to understand, speak, or read English; (10) do-not-resuscitate status; and (11) prior enrollment in this trial. Hereafter we will refer to inclusion and exclusion criteria as inclusion criteria. The primary criterion used to identify potential subjects was an inpatient order for basal-bolus or sliding-scale insulin on one of the units used for recruitment. This criterion was utilized for subject identification as it was expected to be consistent and accurate within the medical record. The identified subjects were then screened for all other criteria.

The research questions, design, outcome measures, and the analytic plan were developed based on numerous face-to-face meetings, telephone calls, and videoconferences with patients with diabetes and with nonprofessional diabetes caregivers, as well as with nurses, physicians, pharmacists, the head of the clinical lab, the Chief Medical Information Officer and other clinical and administrative personnel. Further, we gathered critical insight from thought-leaders within a patient advocacy group, a community group, and professional associations. Then, in preparation for this pilot trial, we performed a retrospective chart review using hospital information systems. From this, we ascertained that 18 % of patients admitted to our hospital have diabetes, and that 85 % of these patients receive subcutaneous insulin while hospitalized (the remainder receive insulin via intravenous infusion). We also ascertained that our population with diabetes has an average length of stay of 6.8 days. We further reviewed the electronic medical records of two random samples of insulin-treated inpatients (N_Total_ = 40) and ascertained that 20 % of these patients would meet inclusion criteria. And finally, we prospectively approached a convenience sample (N = 10) of inpatients meeting inclusion criteria to assess their willingness to participate in such a study. Nine out of the 10 (90 %) patients indicated without hesitation they would enroll, while the tenth patient could not decide immediately.

**Fig. 1:**
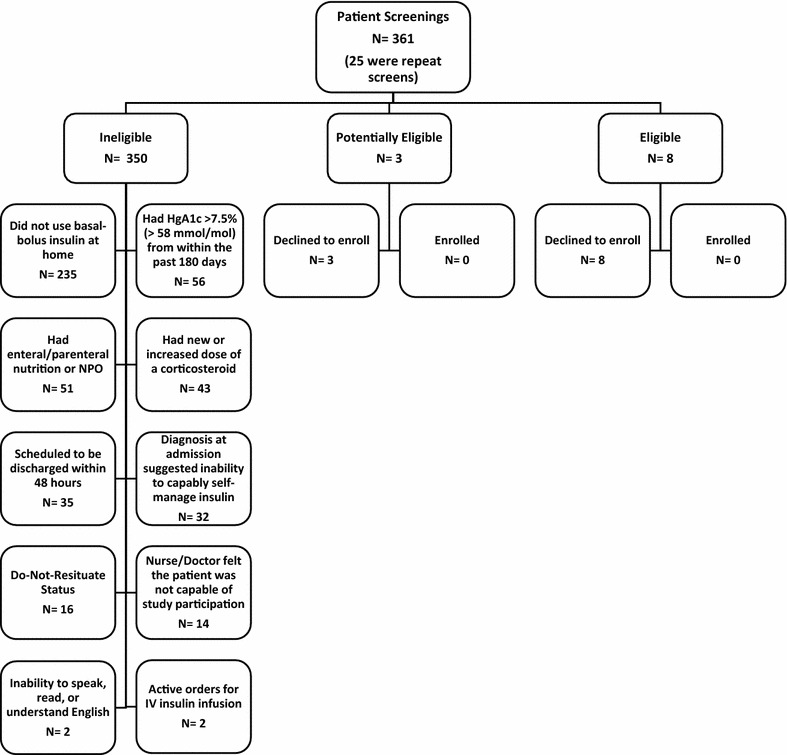
Reasons for non-enrollment

The measured outcomes were feasibility metrics including the number of patients screened, the number of patients deemed eligible, the number of enrolled subjects, the proportion of subjects completing the intervention, and subjects’ satisfaction with their inpatient diabetes care. The study was approved by the University of Pennsylvania’s Institutional Review Board and registered on ClinicalTrials.gov (NCT02144441).

### Results

Operationalizing intervention procedures took approximately 18 months, which was longer than expected. This included obtaining approval from relevant clinical departments, hospital committees, and hospital leadership.

During the 3 months of the study, we performed 361 screenings in 336 patients with orders for basal-bolus or sliding-scale insulin. Repeat screening occurred for patients with multiple admissions. Only 15 screens (4 %) appeared eligible based on chart review. Four of these 15 screens (27 %) were interested in participating but did not meet the inclusion criteria assessed in person. The remaining eleven (73 %) did not wish to enroll. We determined that eight of the eleven subjects (73 %) who did not wish to enroll met all inclusion criteria, while the remaining three (27 %) would have required additional in-person screening to definitively determine eligibility (Fig. 1). The most common reason for declining participation (cited by four of eleven declining patients [36 %]), was lack of interest in being responsible for managing their own insulin therapy given their acute illness.

## Discussion

We hypothesized that allowing eligible hospital inpatients to manage their own insulin could improve the patient-centeredness of care and improve clinical outcomes. We therefore performed a proof-of-concept pilot trial as a precursor to a RCT. Unexpectedly, in contrast to the results of a retrospective chart review and anecdotal evidence obtained from multiple patient and professional sources, we found that very few patients met eligibility criteria, and none of the eight fully eligible subjects or the three potentially eligible subjects were interested in participating. We considered broadening our inclusion criteria, including reducing the anticipated length of stay exclusion criterion from 48 to 24 h and increasing the HbA1c threshold exclusion criterion from 7.5 to 8 %, but decided not to because through interim analyses of screening failures, we found that these minor modifications would likely have had little effect in increasing the number of eligible patients. While extensive consideration was given to altering other criteria, we were concerned about the safety of self-managed insulin in patients not meeting original criteria within the parameters of our pilot design.

We hope that sharing these challenges can advance future research in patient-managed insulin. First, the most common reason for screen failure (65 %) was lack of experience managing basal-bolus insulin. This may be addressable in future studies by including patients whose home regimen is bolus- or basal-only insulin. Additionally, it may be reasonable to limit inclusion to type-1 diabetes patients, who may be better trained in carbohydrate counting and therefore more likely to participate. The resulting reduction in the number of available patients could be accommodated by expanding to multiple centers. Second, given the short duration of typical hospital stays, the eligibility requirement for an expected length of stay of >48 h was responsible for 10 % of screening failures. However, this number probably underestimates the number of screen failures because we sometimes could not ascertain the expected length of stay for patients disqualified for other reasons. The rationale for this criterion was the time needed to educate the patient and the limited opportunity to observe a benefit over short stays. This may be addressable in future studies by including patient care units with longer average lengths of stay. And finally, among the eleven screens eligible for the study, the most common reason for refusal (36 %) was lack of interest in being responsible for their own glucose control during a period of acute illness. Future trials should consider recruiting on units where patients are typically less acutely ill or at skilled nursing facilities rather than hospitals.

Despite guidelines in the U.S. and internationally supporting patient-managed in-hospital insulin [4, 5], there have been no RCTs assessing the efficacy and safety of this approach to care. We sought to assess the feasibility of an RCT by performing a proof-of-concept pilot trial, but found we were not able to enroll any subject using the selected inclusion criteria. The challenges we faced in enrolling patients may be useful to help inform research design and criteria to support future studies into in-hospital patient-managed insulin. Based on our experiences, we would recommend future studies investigate patient self-managed insulin in the hospital by focusing recruitment on patients with type 1 diabetes, on basal-bolus, basal-only or bolus-only regimens at home, who are admitted for non-acute illnesses, within units that typically require longer lengths of stay.

## Abbreviations

RCT: randomized clinical trial
DNP: diabetes nurse practitioner
